# Disrupting ribulose-5-phosphate metabolic flux enhances riboflavin production in *Escherichia coli* BL21(DE3)

**DOI:** 10.1371/journal.pone.0336576

**Published:** 2025-11-14

**Authors:** Junhui Ying, Jin Lu, Qiming Liu, Yingjie Pan, Xianfeng Bao, Junjun Yin, Bing Fu

**Affiliations:** 1 College of Forestry Science and Technology, Lishui Vocational and Technical College, Lishui, Zhejiang, People’s Republic of China; 2 College of Medical Technology, Ningbo College of Health Sciences, Ningbo, Zhejiang, People’s Republic of China; 3 Songyang Rural Industry Development Center, Lishui, Zhejiang, People’s Republic of China; 4 Songyang Agricultural Products Quality and Safety Center, Lishui, Zhejiang, People’s Republic of China; University of the Pacific, UNITED STATES OF AMERICA

## Abstract

Riboflavin (vitamin B_2_) is an essential water-soluble vitamin. To increase its production in a previously engineered strain, R203, we employed metabolic engineering strategies to improve the supply of ribulose-5-phosphate, a key precursor. Disruption of the genes *pfkA* and *edd-eda*, which are aimed at promoting ribulose-5-phosphate generation, increased riboflavin production by 51.27% and 65.81%, respectively. To minimize the consumption of ribulose-5-phosphate, we disrupted *kdsD* and *gutQ*, both of which encode D-arabinose 5-phosphate isomerase. Only the disruption of *gutQ* was effective, increasing production by 19.65%, whereas *kdsD* disruption had no significant effect. Furthermore, disrupting *yajO* and inserting the *pgl* gene increased production by 8.65% and 18.80%, respectively. In contrast, inserting *ribM*, which encodes a riboflavin transporter from *Streptomyces davawensis*, reduced production. The final engineered strain, R19, achieved a riboflavin titer of 2,546.35 ± 159.65 mg/L, representing a 287.35% increase over that of the starting strain. This study provides an effective strategy for high-level riboflavin production in recombinant *Escherichia coli* BL21(DE3) strains.

## Introduction

Riboflavin (RF), i.e., vitamin B_2_, is a water-soluble vitamin belonging to the B vitamins. It was first discovered in milk by the British chemist Alexander Wynter Blyth in 1872 and was officially named in the 1930s [1]. RF has a wide range of physiological functions, but generally does not have a direct metabolic function in cells. RF is usually involved in a wide range of one- and two-electron redox reactions *in vivo* in the form of its derivatives, i.e., flavin mononucleotide (FMN) and flavin adenine dinucleotide (FAD) [2]. In rare cases, RF is also directly involved in redox reactions as a cofactor [3]. RF and its derivatives are indispensable in multiple cellular processes—such as mitochondrial energy metabolism, stress responses, and vitamin and cofactor biogenesis—by serving as cofactors that promote flavoenzyme catalysis, folding, and structural stability [2,4–6]. RF deficiency has been demonstrated to impair the oxidative state of the body, especially in relation to lipid peroxidation status, in both animal and human studies [7]. Furthermore, studies indicate that RF deficiency has profound effects on iron absorption, metabolism of tryptophan, mitochondrial dysfunction, gastrointestinal tract, brain dysfunction and metabolism of other vitamins as well as is associated with skin disorders [8]. Some scholars even suggest that RF deficiency may increase the risk of some cancers [9]. The mechanism for these adverse effects may be that RF deficiency reduces the metabolism of other B vitamins, especially vitamin B_6_ and folic acid [10]. As a result, RF is listed by the World Health Organization as one of the six main indicators for assessing human growth and nutritional status [11].

Currently, *Ashbya gossypii* and *Bacillus* *subtilis* are the most commonly used microorganisms for RF production [11]. As a model organism, *Escherichia coli*, with its clear metabolic background, rapid growth, and mature molecular manipulation techniques, also shows good potential for constructing RF-producing strains [11]. Notably, *E. coli* BL21(DE3) possesses a His115Leu mutation in its FAD synthase (encoded by *ribF*), which favors RF accumulation compared with other *E. coli* strains [12]. Commonly used strategies for the construction of RF-producing engineered bacteria include the following: 1) overexpressing key genes involved in RF synthesis, such as *zwf*, *rib* artificial operon, etc.; 2) reducing metabolic flow to competing pathways by gene knockout or knockdown, such as disruption of *pfkA*, *edd*, *eda*, etc.; 3) decreasing the inhibitory effect on key genes, such as disruption of FMN riboswitch, etc.; and 4) optimizing the culture conditions [5,13–16].

**Fig 1:**
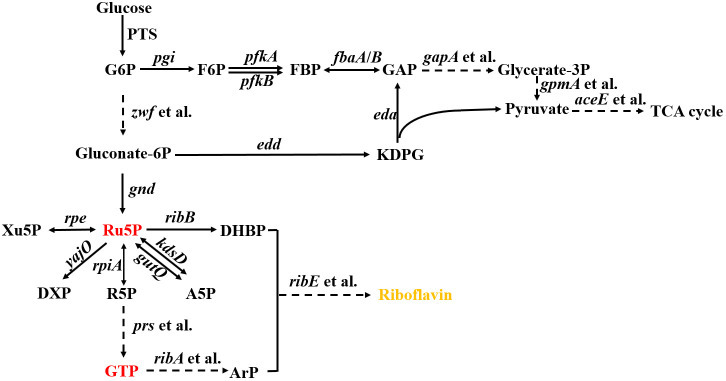
Schematic overview of the relevant pathways for riboflavin synthesis in *E. coli.* The dashed lines indicate multiple enzymatic steps. The names of the genes and the enzymes they encode are as follows: *pgi*, glucose-6-phosphate isomerase; *zwf*, glucose-6-phosphate-1-dehydrogenase; *pfkA*, 6-phosphofructokinase I; *pfkB*, 6-phosphofructokinase II; *edd*, phosphogluconate dehydratase; *eda*, multifunctional 2-keto-3-deoxygluconate 6-phosphate aldolase and 2-keto-4-hydroxyglutarate aldolase and oxaloacetate decarboxylase; *rpe*, ribulose-phosphate 3-epimerase; *yajO*, 1-deoxyxylulose-5-phosphate synthase; *rpiA*, ribose-5-phosphate isomerase A; *kdsD* & *gutQ*, D-arabinose 5-phosphate isomerase; *prs*, ribose-5-phosphate diphosphokinase; *ribA*, GTP cyclohydrolase II; *ribB*, 3,4-dihydroxy-2-butanone-4-phosphate synthase; *ribE*, 6,7-dimethyl-8-ribityllumazine synthase; *fbaA*, fructose-bisphosphate aldolase class II; *fbaB*, fructose-bisphosphate aldolase class I; *gapA*, glyceraldehyde-3-phosphate dehydrogenase; *gpmA*, 2,3-bisphosphoglycerate-dependent phosphoglycerate mutase; *aceE*, pyruvate dehydrogenase. Abbreviations: PTS, phosphoenolpyruvate–carbohydrate phosphotransferase system; G6P, glucose-6-phosphate; F6P, fructose-6-phosphate; Gluconate-6P, gluconate-6-phosphate; DHBP, 3,4-dihydroxy-2-butanone 4-phosphate; ArP, 5-amino-6-(D-ribitylamino)uracil; Ru5P, ribulose-5-phosphate; R5P, ribose-5-phosphate; A5P, arabinose-5-phosphate; DXP, 1-deoxy-D-xylulose-5-phosphate; GTP, guanosine triphosphate; Xu5P, xylulose-5-phosphate; KDPG, 2-keto-3-deoxy-6-phosphogluconate; FBP, fructose 1,6-bisphosphate; GAP, glyceraldehyde 3-phosphate; Glycerate-3P, glycerate-3-phosphate; TCA cycle, tricarboxylic acid cycle.

Increasing the supply of ribulose-5-phosphate (Ru5P) is a key strategy for improving RF production, as Ru5P is both a direct precursor for RF and a precursor for guanosine triphosphate (GTP), another essential RF precursor (Fig 1). Consequently, increasing the intracellular Ru5P concentration promotes RF synthesis. It has been demonstrated that overexpressing *zwf*, *pgl*, and *gnd* channels metabolic flux into the pentose phosphate pathway (PPP), which elevates Ru5P levels and, in turn, increases RF production [14,17].

In addition to the generation of the immediate precursor substance for RF, Ru5P has a variety of other intracellular conversion pathways (Fig 1): 1) interconversion with xylulose-5-phosphate (Xu5P) catalyzed by ribulose-phosphate 3-epimerase (encoded by *rpe*); 2) interconversion with D-arabinose-5-phosphate (A5P), catalyzed by D-arabinose 5-phosphate isomerase (API) [18]; and 3) conversion to 1-deoxy-D-xylulose-5-phosphate (DXP), catalyzed by DXP synthase (encoded by *yajO*).

Disruption of the *rpe* gene strongly affects the growth of *E. coli* [19]. Similarly, in *B. subtilis*, complete inactivation of Rpe activity also resulted in a sharp reduction in the bacterial growth rate, while reducing Rpe enzyme activity through mutation was shown to increase RF production [20]. In *E. coli, kdsD* and *gutQ* encode APIs, a third gene encoding an API (*kpsF*) exists in some pathogenic *E. coli*, and there is well over 40% sequence homology among these three APIs [21]. Disruption of *kdsD* or *gutQ* alone does not affect bacterial growth, but a *gutQ* and *kdsD* double mutant is not viable [22]. Disruption of the *yajO* gene had no effect on the normal growth of the strain [23]. Collectively, these findings suggest that disrupting genes such as *kdsD*, *gutQ*, and *yajO* presents a viable approach to increasing RF production.

**Fig 2:**
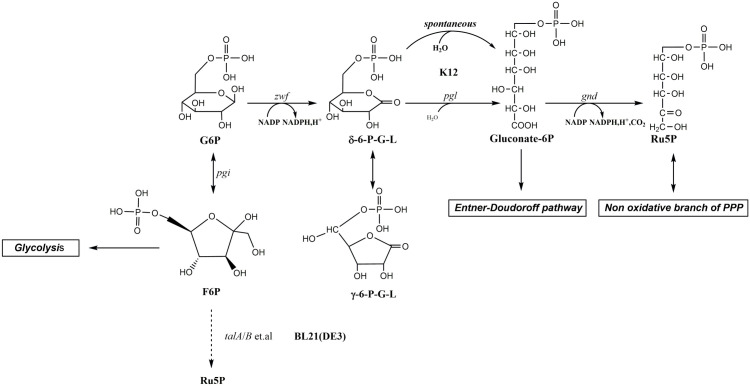
Schematic representation of the differences in the oxidative branch of the PPP between *E. coli* K-12 and BL21(DE3). The dashed lines indicate multiple enzymatic steps. The names of the genes and the enzymes they encode are as follows: *pgl*, 6-phosphogluconolactonase; *talA*/*B*, transaldolase A/B. The names of the other genes and the enzymes they encode are shown in Fig 1. Abbreviations: δ-6-P-G-L, δ-6-phosphogluconolactone; γ-6-P-G-L, γ-6-phosphogluconolactone. Other abbreviations are shown in Fig 1.

Unlike *E. coli* K-12, the gene *pgl* is absent in *E. coli* BL21 (Fig 2). This inevitably leads to a reduction in PPP efficiency and NADPH supply, and a decrease in the supply of Ru5P and erythrose-4-phosphate required for the synthesis of nucleotides and amino acids, as well as the accumulation of γ/δ-6-phosphogluconolactone (γ/δ-6-P-G-L) [24,25]. Therefore, it has been suggested that BL21(DE3) is suboptimal for certain biotechnological applications. Although transaldolase A/B (TalA/B) in BL21(DE3) reconstructed the oxidative pathway of PPP (oxPPP) to a certain extent [26], there is still a need to further strengthen oxPPP by restoring *pgl* to increase RF production.

Oxidative stress is defined as increased generation of reactive oxygen species or a reduced ability to deactivate them, and it is one of the primary factors that induces apoptosis [27,28]. Intracellular flavins (FMN and FAD) and flavoproteins are major sources of the reactive oxygen species ${\text{O}}_{2}^{-}$ and H_2_O_2_, which severely interfere with cellular structure, metabolism and growth [29]. Therefore, promoting RF efflux to reduce the generation of flavins may potentially increase RF production. Although flavins can induce oxidative stress, their precursor, RF, acts as a potential antioxidant by modulating the activity of key antioxidant enzymes such as superoxide dismutase (SOD), catalase, and glutathione peroxidase [8]. Hemberger [30] reported that the expression of the RF transporter gene *ribM* from *Streptomyces davawensis* can improve RF production in *B. subtilis*, suggesting that the enhancement of RF excretion is also a useful strategy for increasing RF production.

As established previously, Ru5P is a key precursor for RF biosynthesis. This study therefore focused on enhancing RF production by employing molecular strategies to engineer the Ru5P synthesis and conversion pathways. The effects of restoring oxPPP and heterologously expressing *ribM* on RF yield were also examined. However, the implications of *kdsD*, *gutQ*, and *yajO* knockouts on RF production remain unexplored. The investigation of these genes could provide valuable insights for further increasing RF yield in engineered *E. coli* strains.

## Materials and methods

### Strains, plasmids, primers, media, and reagents

All strains and expression plasmids used in this study are listed in Table 1. *E. coli* DH5α was used as the host to propagate plasmid DNA, and strain R203, which disrupted the *ribB* FMN riboswitch and completed the *ribF* mutation in *E. coli* BL21(DE3), was used as a parent strain for related genetic modifications [12]. The 4-isopropylbenzoic acid (cumate)-inducible expression vector pNEW-AZ containing six key genes (*ribA*/*B*/*C*/*D*/*E*, and *zwf*) for RF synthesis was constructed by our laboratory previously [12]. The plasmids and primers (including detection primers) for genome editing are listed in S1 and S2 Tables, respectively.

**Table 1:** The strains and plasmids used in this study.

Strains/plasmids	Description	Source/ Reference
**Strains**		
**DH5α**	wild-Type, *F^-^φ80 lac ZΔM15 Δ(lacZYA-arg F) U169 endA1 recA1 hsdR17(rk^-^,mk^+^) supE44λ-thi-1 gyrA96 relA1 phoAe*	Takara
**R203**	BL21(DE3), *ΔsroG*, and *ribF*^(T203D)^	This study
**R6**	R203 containing pNEW-AZ	This study
**R8**	BL21(DE3), *ΔsroG*, *ribF*^(T203D)^, and *ΔpfkA*	This study
**R9**	R8 containing pNEW-AZ	This study
**R10**	BL21(DE3), *ΔsroG*, *ribF*^(T203D)^, *ΔpfkA*, *Δedd*, and *Δeda*	This study
**R11**	R10 containing pNEW-AZ	This study
**R12**	BL21(DE3), *ΔsroG*, *ribF*^(T203D)^, *ΔpfkA*, *Δedd*, *Δeda*, and *ΔkdsD*	This study
**R13**	R12 containing pNEW-AZ	This study
**R14**	BL21(DE3), *ΔsroG*, *ribF*^(T203D)^, *ΔpfkA*, *Δedd*, *Δeda*, and *ΔgutQ*	This study
**R15**	R14 containing pNEW-AZ	This study
**R16**	BL21(DE3), *ΔsroG*, *ribF*^(T203D)^, *ΔpfkA*, *Δedd*, *Δeda*, *ΔgutQ*, and *ΔyajO*	This study
**R17**	R16 containing pNEW-AZ	This study
**BL-1**	BL21(DE3) with pET-AE	Lab stock
**BL-8**	BL21(DE3) with pET-AE & pAC-*pgl*	This study
**R18**	BL21(DE3), *ΔsroG*, *ribF*^(T203D)^, *ΔpfkA*, *Δedd*, *Δeda*, *ΔyajO,* and *purR::pgl*	This study
**R19**	R18 containing pNEW-AZ	This study
**R20**	BL21(DE3), *ΔsroG*, *ribF*^(T203D)^, *ΔpfkA*, *Δedd*, *Δeda*, *ΔyajO, purR::pgl*, and *yghX::ribM*	This study
**R21**	R21 containing pNEW-AZ	This study
**Plasmids**		
**pACYCDuet-1**	expression vector induced by IPTG, two MCS, P_T7-LazO_, Cm^R^, compatible with pETDuet-1	This study
**pAC-*pgl***	pACYCDuet-1 containing *pgl*	This study
**pET-AE**	pETDuet-1 with *ribA*, *ribB*, *ribC*, *ribD*, and *ribE*	This study
**pNEW-AZ**	pNEW containing *ribC, ribE*, *ribB*, *ribD*, *ribA,* and *zwf,* Kan^R^	This study
**pET-23b(+)-*pgl***	pET-23b(+) containing pJ23119, RBS, *pgl*, and T7TΦ	This study
**pET-23b(+)-*ribMopt***	pET-23b(+) containing pJ23119, RBS, optimized *ribM* gene, 6 × His, and T7TΦ	This study

Luria–Bertani (LB) medium composed of 10 g/L tryptone, 5 g/L yeast extract, and 10 g/L NaCl was used for strain propagation and fermentation. Appropriate antibiotics were added to the LB medium when necessary. Unless specifically mentioned, all the strains were incubated in LB at 37 °C under good aeration.

The FastPure plasmid mini kit, FastPure gel DNA extraction mini kit, FastPure^®^ bacteria DNA isolation mini kit, and ClonExpress II one-step cloning kit were purchased from Vazyme Biotech (Nanjing, China); PrimeSTAR^®^ HS (Premix), the competent cell preparation kit, and *Dpn* I restriction endonucleases were purchased from Takara Bio (Dalian, China); the 6-phosphogluconolactonase (6PGLs) enzyme immunoassay kit was purchased from Shanghai C-reagent Biotechnology (Shanghai, China); the BCA protein assay kit, RF standard sample, isopropyl β-D-thiogalactoside (IPTG), and cumate were purchased from Shanghai Biotech (Shanghai, China); Ru5P was purchased from BIOSYNTH (Zurich, Switzerland); A5P was purchased from Cayman Chemical (Michigan, USA); DXP was purchased from Echelon Biosciences (Utah, USA); methanol, formic acid, acetonitrile, and ammonium formate were purchased from McLean Company (Shanghai, China); BeyoLytic™ bacterial active protein extraction reagent was purchased from Beyotime (Shanghai, China); PVDF membrane (0.2 μm) was purchased from Millipore (Massachusetts, USA); ponceau S, Tween-20, PMSF, transmembrane powder, SDS-PAGE electrophoresis powder, and TBST powder were purchased from Servicebio (Wuhan, China); ECL luminescent liquid was purchased from Thermo Fisher Scientific (Massachusetts, USA); anti-6 × His antibody, anti-GAPDH antibody, and goat anti-mouse IgG H&L (HRP) were purchased from Abcam (Cambridge, UK). Primer synthesis and sequencing were performed by Sangon Biotech (Shanghai, China).

### Genetic manipulations

Target DNA fragments were amplified by PCR using a GeneExplorer thermal cycler (Bioer, China) with genomic DNA or plasmids as the templates. Disruption or insertion of relevant genes using CRISPR-Cas9 gene editing technology has been performed as previously described [31]. Reverse PCR was carried out to generate the sgRNA, and overlap PCR was used to obtain the repair templates. A Scient-2C gene transfection instrument (SCIENTZ, China) was used to introduce the sgRNA and repair templates into electroporation-competent cells. The experimental parameters were as follows: voltage, 1500 V; capacitance, 25 μF; resistance, 400 Ω; and gene pulser cuvette, 2 mm [12].

Nucleic acid fragments containing the *pgl* gene from *E. coli* K-12, which encodes 6PGLs, and the constitutive promoter pJ23119 were synthesized by Sangon Biotech (Shanghai, China), and inserted into the vector pET-23(b)+. The resulting plasmid was named pET-23b(+)-*pgl*. This plasmid was used to construct a repair template for subsequent insertion at the *purR* gene locus.

The *ribM* gene from *S. davawensis* which encodes the RF transporter was codon optimized for expression in *E. coli*. The constitutive promoter pJ23119 was used to drive the expression of the target gene, and a 6 × His tag was added for western blot analysis. The above fragment was synthesized by Sangon Biotech (Shanghai, China) and inserted into the vector pET-23(b)+, and the resulting plasmid was named pET-23b(+)-*ribM*_*opt*_. This plasmid was used to construct a repair template for subsequent insertion at the pseudogene *yghX* pseudogene locus.

After gene editing, colony PCR and sequencing were used to screen positive colonies. Subsequently, sgRNA and pCas were sequentially cured by IPTG induction and incubation at 42 °C, respectively.

### Detection methods

The RF concentration was determined by high performance liquid chromatography (HPLC) using an Agilent 1260 LC system (Agilent, USA) as previously described [14].

Ultra performance liquid chromatography tandem mass spectrometry (UPLC–MS/MS) was performed to determine the concentrations of Ru5P, A5P, and DXP. The UPLC analyses were carried out via a UPLC I-Class system (Waters, USA) equipped with an HSS T3 2.1 × 100 mm 1.8 μm column (Waters, USA). The flow rate was set to 0.3 mL/min, and the mobile phase composition was as follows: water-acetonitrile (9:1, v/v).

Crude protein concentration was measured using a BCA kit, and 6PGLs enzyme activity was assayed with a corresponding enzyme immunoassay kit; in both cases, absorbance (562 nm for BCA, 450 nm for 6PGLs) was measured using a TECAN Infinite M200 microplate reader (Tecan, Switzerland), following the manufacturers’ protocols.

The expression of RibM was determined via western blotting, and GAPDH was used as a loading control.

### Statistical analysis

All the experiments were performed in triplicate. The data were processed and analyzed for significance (*P* < 0.05) using SPSS 17.0 software, and the results are presented as the means ± SDs. Figures were graphed using ChemDraw Professional 17.1 and Origin 2018.

## Results

### Effects of *pfkA*, *edd*, and *eda* disruption on RF production

Disruptions of *pfkA*, *edd*, and *eda* have been demonstrated to increase the production of RF in *E. coli* [16]. To further improve the production of RF, *pfkA* was deleted via CRISPR-Cas9 based on the strain BL21-*ΔsroG*-*ribF*^(T203D)^ (R203) which was previously constructed by our laboratory [12]. After screening by colony PCR and sequencing, the positive clone was named R8 (S1 Fig a). Owing to the close genomic localization of *edd* and *eda*, a dual-gene deletion was executed in strain R8 via a single round of CRISPR-Cas9 editing, resulting in strain R10 following validation (S1 Fig b).

**Fig 3:**
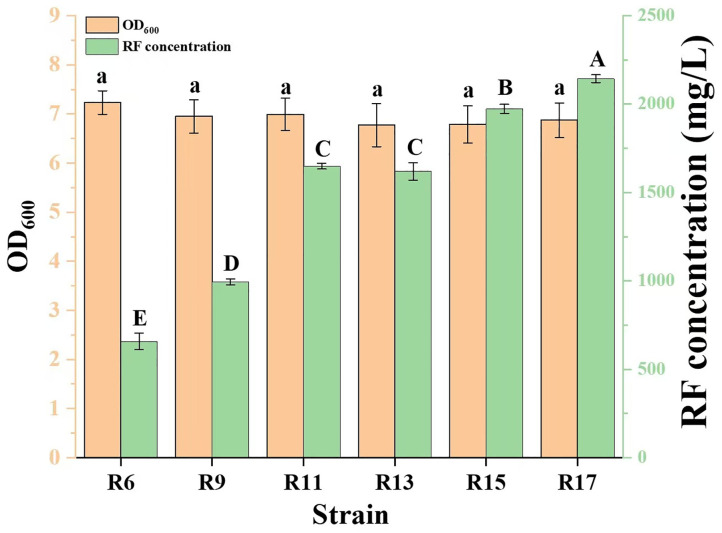
Effect of gene knockouts on RF production. RF concentrations (green bars) and OD_600_ values (orange bars) are shown. Superscript letters indicate significant differences.

The pNEW-AZ plasmid was transformed into strains R8 and R10 competent cells by heat shock, and engineered strains R9 and R11 were obtained, respectively. Strain R9 could accumulate 994.42 ± 16.90 mg/L RF, an increase of 51.27% compared with that of strain R6; strain R11 further increased the RF concentration by 65.81% compared with that of strain R9, which reached 1,648.80 ± 16.17 mg/L (Fig 3). These results suggested that the deletion of *pfkA*, *edd*, and *eda* enhances RF production in *E. coli*, which is consistent with the experimental findings reported by Liu et al [16].

### Effects of disruption of APIs and YajO on intracellular metabolite concentrations and RF production

Using strain R10 as the parent, the *kdsD* and *gutQ* genes were individually knocked out. The resulting strains were verified by colony PCR and sequencing, and were designated as R12 and R14, respectively (S1 Figs c and d). The pNEW-AZ plasmid was then introduced into R12 and R14 to generate the production strains R13 and R15, respectively. Disruption of *kdsD* did not affect RF production, possibly because its isoenzyme, GutQ, compensated for the loss of KdsD function. Moreover, disruption of *gutQ* enhanced RF production by 19.65%, reaching 1,972.71 ± 25.10 mg/L (Fig 3). This finding suggests that the API encoded by *gutQ* possesses greater enzymatic activity than that encoded by *kdsD*. Unfortunately, we were unable to determine the intracellular concentrations of A5P and Ru5P by UPLC-MS/MS due to their isomeric nature. Studies of enzymatic properties may need to be carried out at a later stage using other means. In addition, although disruption of *gutQ* affects the rate of biofilm formation, there are no differences in the growth rate [32].

Strain R16 was constructed by knocking out the *yajO* gene in R14 strain and was verified by colony PCR and sequencing (S1 Fig e). Subsequently, strain R17 was generated by introducing the pNEW-AZ plasmid into R16.Compared with strain R14, strain R16 exhibited a 6.27% decrease in the intracellular DXP concentration (Table 2). Correspondingly, RF production in strain R17 increased by 8.65% over that of strain R15, reaching 2,143.39 ± 21.98 mg/L (Fig 3). The reaction catalyzed by YajO serves as a complementary but low-efficiency pathway for DXP synthesis [33], which explains the limited improvement (8.65%) in RF production upon *yajO* disruption. In *E. coli*, the phosphorylation product of glucose, glucose-6-phosphate, is primarily converted to fructose-6-phosphate (accounting for over 70% of the carbon flux), while only a minor fraction enters the PPP to generate Ru5P [34]. Consequently, disruptions of *gutQ* and *yajO* collectively enhanced RF production by only ∼30%, as their metabolic contributions are constrained by the low Ru5P supply from the PPP. To our knowledge, no prior studies have reported increased RF production by co-deleting *gutQ* and *yajO*, thus offering a novel metabolic engineering strategy.

**Table 2:** Effect of disruption of *yajO* on intracellular DXP concentration.

Strains	DXP (ng/mL)
**RF14**	638.33 ± 2.31^a^
**RF16**	598.33 ± 33.49^b^

Superscript letters indicate significant differences.

### Effect of genomic insertion of *pgl* on RF production

The plasmid pACYCDuet-1-*pgl* (designated pAC-*pgl*) was previously constructed in our laboratory. To validate the effect of *pgl* expression, pAC-*pgl* was transformed into BL-1 [14] competent cells harboring the plasmid pETDuet-1-*ribA-ribB-ribC-ribD-ribE* (pET-AE), generating the engineered strain BL-8. Upon induction with 2 mM IPTG, compared with strain BL-1, strain BL-8 presented a 35.34% increase in RF production and a 14.73% increase in the OD_600_ (Table 3). In contrast, co-expression of *pgl*, *zwf*, and *gnd* in *E. coli* K-12 increased RF production by 18.9% [17]. These results indicate that *pgl* expression in *E. coli* BL21(DE3) more effectively enhances RF production than in *E. coli* K-12. Additionally, *pgl* expression improved the cellular growth rate. Given that the oxPPP serves as the primary source of NADPH, *pgl* expression in *E. coli* BL21(DE3) concomitantly enhanced both RF biosynthesis and biomass accumulation.

**Table 3:** Effect of overexpression of *pgl* on bacterial growth and RF production.

Strains	OD_600_	RF production (mg/L)
**BL-1**	7.13 ± 0.13^b^	182.65 ± 9.04^B^
**BL-8**	8.18 ± 0.22^a^	247.19 ± 26.09^A^

Superscript letters indicate significant differences.

To restore oxPPP, the *pgl* gene was inserted via CRISPR-Cas9 in strain R16, generating strain R18 (S1 Fig f). An activity of 11.42 ± 0.98 U/g·L of 6PGLs was detected in the crude enzyme solution of strain R18, whereas no 6PGL activity was detected in that of strain R16 (Table 4), confirming successful *pgl* expression in strain R18. To evaluate the effect of *pgl* chromosomal integration on RF production, pNEW-AZ was transformed into R18 competent cells, yielding strain R19. Compared with that of strain R17, the RF production of strain R19 was effectively increaseed to 2,546.35 ± 159.65 mg/L, which was an 18.80% increase; the insertion of the *pgl* gene also increased the biomass of the strain, and the OD_600_ value of strain R19 was increased by 7.42% compared with that of strain R17 (Table 5). Additionally, inserting the *pgl* gene into the *purR* locus also led to inactivation of the PurR regulator, which could be another reason for the increase in RF yield.

**Table 4:** Comparison of crude protein 6PGLs activity of different strains.

Strains	6PGLs activity (U/g·L)
**R16**	N/D
**R18**	11.42 ± 0.98

N/D indicates no 6PGLs activity was detected.

**Table 5:** Effect of *pgl* insertion on bacterial growth and RF production of strains.

Strains	OD_600_	RF production (mg/L)
**R17**	6.87 ± 0.35^b^	2,143.39 ± 21.98^B^
**R19**	7.38 ± 0.56^a^	2,546.35 ± 159.65^A^

Superscript letters indicate significant differences.

### Effect of heterologous expression of *ribM* on RF production

**Fig 4:**
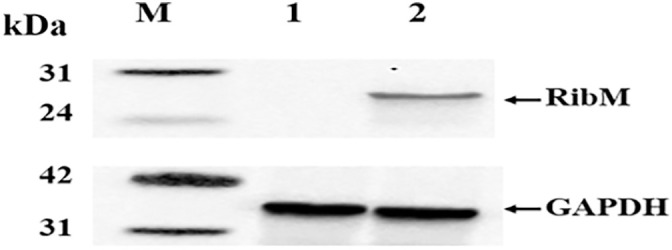
Result of western blot for detecting the expression of RibM. Lane M: StarSignal Western Protein Marker; lane 1: total soluble protein of strain R18; lane 2: total soluble protein of strain R20.

The *ribM* gene from *S. davawensis* was inserted into the *yghX* locus of strain R18 by CRISPR-Cas9 to obtain strain R20 (S1 Fig g). The successful expression of the RibM protein in strain R20 was demonstrated by western blot experiments, which detected a 24.7 kDa target band (containing a 6 × His tag) (Fig 4).

There was no difference between the OD_600_ values of strains R18 and R20 (∼6.80), indicating that the heterologous expression of optimized *ribM* in *E. coli* was not toxic to the host bacteria. The RibM derived from *S. davawensis* does not consume ATP when RF is transported, and its expression in *B. subtilis* has been confirmed to increase RF production [30]. In contrast to the 18% improvement achieved by Hemberger et al. [30] through *ribM* expression in *B. subtilis*, a negative result was presented for the R21 strain (Table 6), which is likely caused by an increased metabolic burden, among other factors [35]. Although the RF concentration in the cell lysates of strain R21 was lower than that in the lysates of strain R19 (Table 6), it remains unclear whether this reduction was due to active export by RibM. Therefore, follow-up experiments are needed to explain the decrease in RF yield.

**Table 6:** Effect of *ribM* insertion on bacterial growth and RF concentration in intracellular and fermentation broths.

Strains	OD_600_	RF concentration in cell lysates (mg/L)	RF concentration in fermentation broth (mg/L)
**R19**	7.38 ± 0.56^a^	7.38 ± 0.56^a^	2,546.35 ± 159.65^A^
**R21**	4.80 ± 0.23^b^	6.40 ± 1.32^b^	945.68 ± 44.62^B^

Superscript letters indicate significant differences.

## Discussion

RF is widely used in pharmaceuticals, cosmetics, food production, animal husbandry, and various other sectors. The global RF market was valued at an estimated $13.46 billion in 2022 and is projected to reach $23.34 billion by 2031 [36]. Therefore, enhancing RF production is crucial to meet the growing demand.

RF and its derivatives play crucial roles in numerous biological processes, including protein folding, electron transfer in the respiratory chain, nucleic acid synthesis, DNA photorepair, the synthesis of natural therapeutic products, the regulation of apoptosis independent of apoptotic proteases, and the promotion of glucose metabolism, lipid metabolism, protein metabolism, and growth and development [2,4]. Many microorganisms, including *Campylobacter jejuni*, *Shewanella*, and *Geothrix fermentans*, secrete extracellular RF, which acts as an electron shuttle to enhance iron reduction [37]. In addition, FAD stabilizes the clock protein cryptochrome, modifying rhythmic clock gene expression [38]. RF and FMN serve as primary and secondary emitters in bacterial luminescence [39].

Owing to the lack of the RF biosynthesis pathway in the human body, RF absorption mainly occurs in a specific manner, i.e., through the human RF transporter-mediated trans-polarized enterocyte brush border membrane and basolateral membrane. This process differs significantly from that in pathogenic microorganisms. Therefore, key enzymes, transporter proteins, and FMN riboswitches in the pathogenic microbial RF biosynthesis pathway can be used as potential targets for the development of novel, safe, and effective antimicrobial and anti-infective drugs. For example, 2,6-dioxo-(1H,3H)-9-N-ribosylpurine and 2,6-dioxo-(1H, 3H)-8-aza-9-N-ribosylpurine can inhibit the activities of lumazine synthase (encoded by *ribE*) and RF synthase (encoded by *ribC*), and thus can be candidates for novel antibacterial substances [40].

Ru5P is a pivotal intermediate in the PPP, contributing to cellular energy metabolism, nucleotide synthesis, and redox homeostasis [18]. It is also an intermediate in carbon fixation in photosynthetic organisms and is involved in the biosynthesis of lipopolysaccharides, amino acids, secondary metabolites, and antibiotics [34]. As a key precursor for RF synthesis, elevating the concentration of Ru5P is beneficial for increasing RF production. Strategies involving the overexpression of key genes such as *zwf* and *pgl*, or the knockout of non-essential metabolic pathways to increase Ru5P production, have been successfully implemented in the construction of diverse RF-producing microbial strains [16,17,41,42]. In contrast to the aforementioned studies that focused on promoting the production of Ru5P, this study aimed to reduce its consumption by knocking out *gutQ* and *yajO*. Supporting this approach, Yang et al. [20] also achieved the goal of reducing Ru5P consumption and enhancing RF production by decreasing the enzymatic activity of Rpe in *B. subtilis* LY.

Lipopolysaccharide, commonly known as endotoxin, is a major constituent of the outer membrane in Gram-negative bacteria, including *E. coli* [43]. Endotoxins are difficult to remove and cause severe biological effects, such as fever and septic shock, in mammals [44]. These inherent risks limit the use of *E. coli* in the production of therapeutic recombinant proteins and other related products. Since *kdsD* and *gutQ* are essential for the biosynthesis of 3-deoxy-D-manno-oct-2-ulosonic acid, a characteristic component of bacterial endotoxin, disruption of these genes has been used to construct detoxified *E. coli* strains [45]. Therefore, knockout of the *gutQ* gene contributes to the construction of an endotoxin-free RF-producing strain, enabling the use of engineered *E. coli*-derived RF in clinical and other medical applications. In addition, Wang et al. [46] reported that chromosomal integration and expression of HPV L1 protein genes at the *gutQ*, *kdsD*, and other gene loci provide a viable strategy for obtaining a low-endotoxin HPV 9-valent vaccine. In *E. coli*, the overexpression of *yajO* enhances the terpene yield of engineered bacteria [47]. Ko et al. [23] reported elevated levels of acetol and 1,2-propanediol in a *yajO* disruption strain compared with the wild type.

Elevating the expression of the *pgl* gene in *E. coli* has been shown to promote RF production. In *E. coli* MG1655, Lin et al. [17] overexpressed the *pgl* gene using the p15Trc plasmid, and the resulting strain presented an 18.9% increase in RF production. Zhang et al. [48] inserted the tac promoter-driven *pgl* gene into the *E. coli* MG1655 pseudogene *yghX*, and RF production was increased by 5.2%. The BL-8 strain constructed in this study, which expressed the *pgl* gene by plasmid, enhanced RF production by 35.34%, whereas the R19 strain, which expressed the *pgl* gene on the genome, enhanced RF production by 18.80%. Our results exceed those reported by both teams, suggesting that *pgl* expression in the *pgl*-deficient BL21(DE3) strain might be more effective for enhancing RF production than that in strains such as *E. coli* MG1655. This finding also indicates that to construct genetically engineered strains based on BL21(DE3) for the production of compounds such as aromatic amino acids and vitamins, overexpression of *pgl* is necessary.

The heterologous expression of the *ribM* gene resulted in an adverse effect, potentially due to an increased metabolic burden. However, the mechanisms underlying metabolic burden are complex and interconnected [35]. Strategies to mitigate metabolic burden include, but are not limited to, metabolic balancing, enhancing respiration, dynamic regulatory systems, chromosomal engineering, decoupling cell growth from production phases, and the co-utilization of nutrient resources [49,50]. Furthermore, as RibM is a membrane protein, its correct localization may also be critical [30]. Promoting RF secretion and reducing oxidative stress remain potential strategies to increase RF production, but the regulation of RF accumulation and secretion into the culture medium requires further investigation [4,11,51]. The expression of Dodecin from *S. davawensis*, may serve as an effective alternative strategy to reduce oxidative stress and increase RF production [29].

Researchers have constructed genetically engineered strains for RF production based on K- and B-series *E. coli* strains. Using *E. coli* K-12 MG1655 as the host, Tao Chen’s group developed the engineered strains RF18S [52], LS02T [16], RF05S-M40 [17], LS72T [13], and WY40 [15]. Under shake-flask culture conditions in non-optimized media, 387.6, 667, 1,036.1, 1,339, and 1,454.5 mg/L RF were produced, respectively. In addition, Zhang et al. [48] constructed an antibiotic-free strain, EF18, with a shake flask culture capable of producing 1,287 mg/L RF. Based on *E. coli* BL21(DE3), our laboratory constructed engineered strains R4 [14] and R6 [12], which produced 437.58 ± 14.36 and 657.38 ± 47.48 mg/L RF, respectively. Compared with commercial RF-producing strains, engineered strains developed based on *E. coli* still have room for further improvement in terms of production [11]. However, most of these methods use glucose as the sole carbon source [11]. To reduce production costs, it is essential to conduct research related to mixed sugar fermentation at a later stage.

In this study, we performed sequential gene edits using strain R203 as the parent strain. The resulting strain, R19, achieved a RF titer of 2,546.35 ± 159.65 mg/L, representing a 287.35% increase over R6. To our knowledge, this constitutes the highest RF titer reported under unoptimized medium conditions. Building on this finding, our subsequent work will focus on optimizing the culture parameters for R19 and elucidating the regulatory mechanisms governing RF accumulation and secretion. Furthermore, since Ru5P is a precursor to erythrose-4-phosphate, the strategies developed here may also offer valuable insights for the metabolically engineered production of aromatic amino acids, such as phenylalanine, tyrosine, and tryptophan.

## Conclusion

Overall, metabolic engineering modifications targeting Ru5P effectively enhanced RF production. Specifically, disrupting the *pfkA*, *edd*, and *eda* genes significantly increased RF yield. Disruption of *kdsD* had no effect, likely due to functional compensation by its isozyme GutQ; conversely, disrupting *gutQ* improved RF yield. Disruption of *yajO* similarly enhanced RF production, although the modest catalytic efficiency of its encoded enzyme resulted in only a marginal improvement. Inserting the *pgl* gene into the *purR* locus proved beneficial for both RF yield and bacterial growth. However, the constitutive expression of *ribM* adversely affected both RF yield and growth.

## Supporting information

S1 Fig Electrophoresis of colony PCR verification for different genetically engineered bacterial strains. a. Lanes: M1, DL 5000 DNA marker; M2, DL 2000 DNA marker; 1, colony PCR product of strain R203; 2, colony PCR product of strain R8. b. Lanes: M1, DL 5000 DNA marker; M2, DL 2000 DNA marker; 1, colony PCR product of strain R8; 2, colony PCR product of strain R10. c. Lanes: M1, DL 5000 DNA marker; M2, DL 2000 DNA marker; 1, colony PCR product of strain R10; 2, colony PCR product of strain R12. d. Lanes: M1, DL 5000 DNA marker; M2, DL 2000 DNA marker; 1, colony PCR product of strain R12; 2, colony PCR product of strain R14. e. Lanes: M1, DL 5000 DNA marker; M2, DL 2000 DNA marker; 1, colony PCR product of strain R14; 2, colony PCR product of strain R16. f. Lanes: M1, DL 5000 DNA marker; M2, DL 2000 DNA marker; 1, colony PCR product of strain R16; 2, colony PCR product of strain R18. g. Lanes: M1, DL 5000 DNA marker; M2, DL 2000 DNA marker; 1, colony PCR product of strain R18; 2, colony PCR product of strain R20.
(TIF)

S1 File Raw_image-6 × His. Western blot analysis of uncropped images showing 6 × His-tagged protein expression in different engineered strains. (TIF)

S2 File Raw_image-GAPDH. Western blot analysis of uncropped images showing GAPDH expression in different engineered strains. (TIF)

S1 Table Plasmids used for CRISPR-Cas9 gene editing in this study. (DOCX)

S2 Table Primers used for CRISPR-Cas9 gene editing in this study. (DOCX)
