# Supplementary material for: Disrupting ribulose-5-phosphate metabolic flux enhances riboflavin production in Escherichia coli BL21(DE3)
Source: PLoS One. 2025 Nov 14;20(11):e0336576. doi: 10.1371/journal.pone.0336576 (PMC12617950; doi:10.1371/journal.pone.0336576)
Supplement: S1 Table — (DOCX) [file pone.0336576.s004.docx]

**S1 Table. Plasmids used for CRISPR-Cas9 gene editing in this study.**

| **Strains/plasmids** | **Description** | **Source/**  **Reference** |
| --- | --- | --- |
| **pCas** | *repA101(Ts) kan Pcas-cas9 ParaB-Red lacIq Ptrc-sgRNA-pMB1* | Lab stock |
| **pTargetF** | *pMB1 aadA sgRNA-cadA* | Lab stock |
| **sgRNA-*pfkA*** | *pMB1*, *aadA*, sgRNA target to the *pfkA* of BL21(DE3) | This study |
| **sgRNA-*edd*-*eda*** | *pMB1*, *aadA*, sgRNA target to the *edd* and *eda* of BL21(DE3) | This study |
| **sgRNA-*kdsD*** | *pMB1*, *aadA*, sgRNA target to the *kdsD* of BL21(DE3) | This study |
| **sgRNA-*gutQ*** | *pMB1*, *aadA*, sgRNA target to the *gutQ* of BL21(DE3) | This study |
| **sgRNA-*yajO*** | *pMB1*, *aadA*, sgRNA target to the *yajO* of BL21(DE3) | This study |
| **pET-23b(+)-*pgl*** | pET-23b(+) containing *pJ23119*, RBS, *pgl*, and T7TΦ | This study |
| **sgRNA-*purR*** | *pMB1*, *aadA*, sgRNA target to the *purR* of BL21(DE3) | This study |
| **pET-23b(+)-*ribM_opt_*** | pET-23b(+) containing *pJ23119*, RBS, optimized *ribM* gene，6×His, and T7TΦ | This study |
| **sgRNA-*yghX*** | *pMB1*, *aadA*, sgRNA target to the *yghX* of BL21(DE3) | This study |
