# Supplementary material for: Disrupting ribulose-5-phosphate metabolic flux enhances riboflavin production in Escherichia coli BL21(DE3)
Source: PLoS One. 2025 Nov 14;20(11):e0336576. doi: 10.1371/journal.pone.0336576 (PMC12617950; doi:10.1371/journal.pone.0336576)
Supplement: S2 Table — (DOCX) [file pone.0336576.s005.docx]

**S2 Table. Primers used for CRISPR-Cas9 gene editing in this study.**

| **Primers** | **Sequence (5′→3′)** |
| --- | --- |
| ***pfkA*-sgRNA-F** | TTACAGCGTTTCTGACATGAGTTTTAGAGCTAGAAATAGC |
| ***pfkA*-sgRNA-R** | TCATGTCAGAAACGCTGTAAACTAGTATTATACCTAGGAC |
| ***pfkA*-up-F** | CAGGGCCGACCCGCTTTATTCAG |
| ***pfkA*-up-R** | GCCTTTTTCCGAAATCAGACTACCTCTGAACTTTGGAATG |
| ***pfkA*-down-R** | CCACTTGTTCATCGCCCGACTCT |
| ***pfkA*-down-F** | CAAAGTTCAGAGGTAGTCTGATTTCGGAAAAAGGCAGATT |
| ***pfkA*-JC-F** | GTGTATAAAATACCGCCATTTGGCCTGAC |
| ***pfkA*-JC-R** | CCTGATAAGCGAAGCGCATCAGG |
| ***edd*-sgRNA-F** | ACCTGAGCGCGTCACGCGTGGTTTTAGAGCTAGAAATAGC |
| ***edd*-sgRNA-R** | CACGCGTGACGCGCTCAGGTACTAGTATTATACCTAGGAC |
| ***edd-eda*-up-F** | TGCGCAGGAGATTGCCCGTAAAG |
| ***edd-eda*-up-R** | CAATTTCAGGAGCCTTTAAGTCAAAACGCCCGATCTAC |
| ***edd-eda*-down-F** | GTAGATCGGGCGTTTTGACTTAAAGGCTCCTGAAATTG |
| ***edd-eda*-down-R** | CCTTTAATCAGACGCATCTGGCGGATGC |
| ***edd-eda*-JC-F** | GTTGACGCCATTGAACGCG |
| ***edd-eda*-JC-R** | CCCTATGAGCTCCGGTTACAGG |
| ***kdsD*-sgRNA-F** | GATTGAACGTGAATGCCTGGGTTTTAGAGCTAGAAATAGC |
| ***kdsD*-sgRNA-R** | CCAGGCATTCACGTTCAATCACTAGTATTATACCTAGGAC |
| ***kdsD*-up-F** | GACGGCAATTGCTATCGGAACCA |
| ***kdsD*-up-R** | TTGTTTATCCTTGAATCTAATGCGTTTCCAGTTATTCAAC |
| ***kdsD*-down-F** | AATAACTGGAAACGCATTAGATTCAAGGATAAACAACAAT |
| ***kdsD*-down-R** | TGTGGCACAACGATCTTCTACCA |
| ***kdsD*-JC-F** | TGACGGCAATTGCTATCGGAACCA |
| ***kdsD*-JC-R** | TGTGGCACAACGATCTTCTACCA |
| ***gutQ*-sgRNA-F** | AAATCATCGCCCAGACGTTCGTTTTAGAGCTAGAAATAGC |
| ***gutQ*-sgRNA-R** | GAACGTCTGGGCGATGATTTACTAGTATTATACCTAGGAC |
| ***gutQ*-up-F** | GTTTTGCAGATGGTGCCCCTGC |
| ***gutQ*-up-R** | CTGGGATTGAAGGATTAATCACTCATTGCTCTCTCCGGTTAT |
| ***gutQ*-down-F** | ATAACCGGAGAGAGCAATGAGTGATTAATCCTTCAATCCCAG |
| ***gutQ*-down-R** | GACTAACCGCGATTTACGCGAAGAGG |
| ***gutQ*-JC-F** | GCTGGCAGAGAATGCCTTC |
| ***gutQ*-JC-R** | GCGAACTGGAACATGCTATTCATC |
| ***yajO*-sgRNA-F** | CGCGTTTCCCGACTTTGCCTGTTTTAGAGCTAGAAATAGC |
| ***yajO*-sgRNA-R** | AGGCAAAGTCGGGAAACGCGACTAGTATTATACCTAGGAC |
| ***yajO*-up-F** | GTGATGCTGGGGATCTGTATCGG |
| ***yajO*-up-R** | GGCCCATCGTAAACCAGTACCCG |
| ***yajO*-down-F** | TTATTTAAATCCTACGACCGC |
| ***yajO*-down-R** | ATGCAATACAACCCCTTAGGA |
| ***purR*-sgRNA-F** | GAATCAGGGTGTAACCTTTCGTTTTAGAGCTAGAAATAGC |
| ***purR*-sgRNA-R** | GAAAGGTTACACCCTGATTCACTAGTATTATACCTAGGAC |
| ***purR*::*pgl*-up-F** | ATGCCGCAGGGTACGCCAGTGCTTTCAGT |
| ***purR*::*pgl*-up-R** | ACCTAGGACTGAGCTAGCTGTCAAGCTTTTCCAGTTTCGG |
| ***purR*::*pgl*-mid-F** | ATCCGAAACTGGAAAAGCTTGACAGCTAGCTCAGTCCTA |
| ***purR*::*pgl*-mid-R** | GCGAGAGGCTTTTCAAAAAACCCCTCAAGACCCGTTTAGAGG |
| ***purR*::*pgl*-down-F** | TCTAAACGGGTCTTGAGGGGTTTTTTGAAAAGCCTCTCGCGAG |
| ***purR*::*pgl*-down-R** | CCGTCTGGACGTTCAGTTCGACAATGTGGAAGAAGCTATTGCC |
| ***purR*::*pgl-*JC-F** | CGTCAAGATCGGCCAAAATTCCACGC |
| ***purR*::*pgl-*JC-R** | CTGGGAATCATCGGCAACCTGTATCTCG |
| ***yghX*-sgRNA-F** | CCTTTGCCAGGAAAGATCGGGTTTTAGAGCTAGAAATAG |
| ***yghX*-sgRNA-R** | CCGATCTTTCCTGGCAAAGGACTAGTATTATACCTAGGAC |
| ***yghX*::*ribM*-up-F** | GCGATCGATGCGCCATTTACCACAC |
| ***yghX*::*ribM*-up-R** | TACCTAGGACTGAGCTAGCTGTCAATAGGTTTATCTCTTA |
| ***yghX*::*ribM-*mid-F** | CGTAAGAGATAAACCTATTGACAGCTAGCTCAGTCCTAGGTA |
| ***yghX*::*ribM*-mid-R** | GTTGATCCAACCAAACTGCAAAAAACCCCTCAAGA |
| ***yghX*::*ribM*-down-F** | TCTTGAGGGGTTTTTTGCAGTTTGGTTGGATCAAC |
| ***yghX*::*ribM*-down-R** | CGCGCTTATGCTTTGCTTAAAAAAACACCAGT |
| ***yghX*::*ribM*-JC-F** | GTCACATTAACCGCACTGTCGGC |
| ***yghX*::*ribM*-JC-R** | CCGAAATTGTGCCATAAACCGAGCG |
